# Improvement of the selectivity of isophorone hydrogenation by Lewis acids

**DOI:** 10.1098/rsos.171523

**Published:** 2018-05-23

**Authors:** Yucui Hou, Shuhang Ren, Muge Niu, Weize Wu

**Affiliations:** 1 Department of Chemistry, Taiyuan Normal University, Jinzhong 030619, People's Republic of China; 2 State Key Laboratory of Chemical Resource Engineering, Beijing University of Chemical Technology, Beijing 100029, People's Republic of China

**Keywords:** hydrogenation, selectivity, isophorone, Lewis acid, mechanism

## Abstract

The selective hydrogenation of isophorone (3,5,5-trimethyl-2- cyclohexen-1-one) to produce 3,3,5-trimethylcyclohexanone (TMCH), an important organic solvent and pharmaceutical intermediate, is of significance in industry. However, the over-hydrogenation to produce the by-product 3,3,5-trimethylcyclohexanol causes issues. Up to now, it is still a challenge to hydrogenate isophorone to TMCH with high selectivity. In this work, we found that Lewis acids could inhibit the hydrogenation of C=O bond on isophorone, thus greatly improving the selectivity towards TMCH. In addition, added solvents like supercritical CO_2_ also had a positive impact on the selectivity. Both the conversion and selectivity could be increased to more than 99% when suitable Lewis acid and solvent were employed. Nevertheless, Lewis acid also exhibited some inhibition on the hydrogenation of the C=C bond of isophorone. Hence, a relatively weak Lewis acid, ZnCl_2_, is suitable for the selective hydrogenation.

## 1. Introduction

**Scheme 1.**
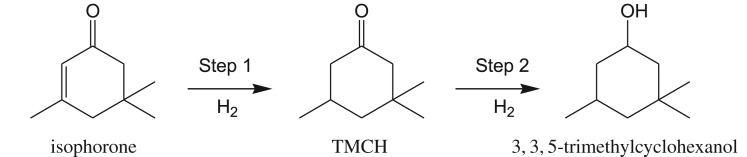
The main reaction steps of isophorone hydrogenation.

3,3,5-Trimethylcyclohexanone (TMCH) is an important pharmaceutical intermediate and can be used as a solvent for vinyl resins, lacquers, varnishes, paints and other coatings [1–5]. It is produced in the industry mainly via the hydrogenation of isophorone (3,5,5-trimethyl- 2-cyclohexen-1-one). Isophorone has a C=C bond and a C=O bond on its molecular structure. The main reaction pathway of isophorone hydrogenation is shown in scheme 1. Conventional processes of the hydrogenation of isophorone produce a low overall conversion with a high selectivity to TMCH or a high overall conversion with poor selectivity for the over-hydrogenation to 3,3,5-trimethylcyclohexanol [4,6]. Owing to the difficult separation between TMCH and 3,3,5-trimethylcyclohexanol for their very close boiling points (189°C and 194–198°C, respectively), a process that combines high conversion and high selectivity to TMCH becomes very much desirable [4,7]. Therefore, a highly selective and efficient process is required for the hydrogenation of isophorone [8–10].

To improve the hydrogenation reaction, some modified catalysts and new reaction solvents were employed. Pisarek *et al*. [11] found that by using the catalyst Ni-Al alloy modified with Cr, a conversion of 63% and a selectivity of 83% were obtained at 80°C. The catalyst Raney Ni modified with Cr on hydrogenation of isophorone was studied and it was found that further hydrogenation of TMCH was restrained by the Cr-containing catalysts [11]. Sato *et al*. [7] reported that the hydrogenation of isophorone, using supercritical carbon dioxide as a solvent with noble metal catalysts, could also achieve an improved selectivity. Overall, the hydrogenation of isophorone undergoes two kinds of hydrogenation, for C=C bond and C=O bond. The hydrogenation of C=C bond is relatively easier to occur than that of C=O bond according to the laws of thermodynamics.

Pd catalyst has a tendency to catalyse the hydrogenation of C=C and C=O [12]. As a commercially available catalyst, activated carbon supported palladium (Pd/AC) could activate H_2_ and showed a high ability for hydrogenation [13,14]. Liu *et al*. found that Lewis acid could interact with carbonyl group on cyclohexanone and protect this group from further hydrogenation over Pd/AC [14]. In the light of their research, it is possible that the inhibitory effect of the Lewis acid can occur on the hydrogenation of the C=O bond in the hydrogenation reaction of isophorone. Therefore, we expect that the by-product of isophorone hydrogenation reaction, 3,3,5-trimethylcyclohexanol, could be inhibited with a Lewis acid.

In this work, several Lewis acids were examined on the hydrogenation of isophorone to TMCH over Pd/AC. It was found that solid Lewis acid in the reaction did exhibit an inhibition of the hydrogenation of the carbonyl group on isophorone molecules. The results showed that Lewis acid also had an inhibition of the hydrogenation of the C=C bond, and the intensity of the inhibition by different Lewis acids was different. As a relatively weak Lewis acid, ZnCl_2_ has a weak inhibition of the hydrogenation of the C=C bond, while effectively inhibiting the over-hydrogenation of the carbonyl group. In addition, some reaction solvents, such as CH_2_Cl_2_ and supercritical CO_2_, also play a similar role as Lewis acid to inhibit over-hydrogenation. The mechanism of the Lewis acid's inhibition was studied, and it indicates that Lewis acid can interact with carbonyl group and protect it against the over-hydrogenation.

## 2. Experimental procedure

### 2.1. Materials

Isophorone (97%) and zinc chloride (more than 98%) were purchased from Aladdin Chemical Reagent Inc. (Shanghai, China). Ethanol (greater than 99.7%) was purchased from Beijing Chemical Plant (Beijing, China). TMCH (98%) and 3,3,5-trimethylcyclohexanol (greater than 90.0%) were purchased from Tokyo Chemical Co., Ltd (Tokyo, Japan). Copper chloride (greater than 99.0%), chromium chloride (greater than 99.0%) and aluminium chloride (greater than 99.0%) were purchased from Tianjin Fuchen Chemical Co., Ltd (Tianjin, China). Pd/AC catalyst (5 wt% of Pd, carbon supported) was supplied by the Research Institute of Petroleum Processing, SINOPEC CORP. (Beijing, China). H_2_ (99.99%), CO_2_ (99.99%) and N_2_ (99.99%) were provided by Beijing Haipu Gases Co., Ltd (Beijing, China). All reagents and solvents were of analytical grade and used without further purification.

### 2.2. Apparatus and procedures

The hydrogenation of isophorone was carried out in a high-pressure batch reactor made from Hastelloy alloy (HC 276), supplied by Haian Petroleum Scientific Research Co., Ltd, Jiangsu, China. The inner volume of the reactor was 25 cm^3^ (20 mm in inner diameter and 79 mm in height) and a magnetic stirrer was used to mix the reactants inside. Typically, 0.6 g isophorone, 60 mg Pd/AC catalyst, 60 mg Lewis acid and the magnetic stirrer were added in the reactor, air in the reactor was purged by hydrogen, and then hydrogen was charged into the reactor to a desired pressure, which was monitored by a pressure gauge composed of a pressure transducer (KLP-800KG) and an indicator (Beijing Tianchen Instrument Co., Ltd, China). After that, the reactor was submerged into a heating furnace, whose temperature was already increased to a desired value. Then the reactor was heated up at a rate of 8–10°C min^−1^ to a desired reaction temperature controlled by a temperature controller (XTD-7000) and monitored by a K-type thermocouple within an accuracy of ±1°C. During the reaction, the mixture was stirred by a magnetic stirrer at a constant speed of 500 r.p.m. After the reaction, the reactor was transferred into a cold water bath for rapid cooling to stop further reaction. Then the mixture remaining in the reactor was transferred into a beaker and the catalyst was filtrated. After that, the filtrate was diluted by solvent, such as ethanol or dichloromethane, before the GC (gas chromatography) analysis. Three parallel experiments were performed at each set of conditions, and the results reported herein represent the mean values. A gradient elution procedure was used for the GC (GC-2014, Shimadzu, Japan) analysis of the reaction products. The mobile phase was N_2_ and the stationary phase was a capillary column (DB-Wax, Agilent, USA). A FID (flame ionization detector) was used for the quantification of the products. Isophorone and the products were identified by a contrast of the retention time with standard substances. The mechanism of hydrogenation of isophorone to TMCH by Lewis acids was studied using Fourier transform infrared spectrometer (Nicolet 6700, Thermo Nicolet Scientific, USA).

## 3. Results and discussion

Table 1 shows the results of the hydrogenation reaction over Pd/AC catalyst in the absence of solvent with or without Lewis acids. When only the Pd/AC catalyst was used (table 1, entry 1), the conversion of isophorone was very high (99.9%). However, because most of isophorone was over-hydrogenated to 3,3,5-trimethylcyclohexanol, the selectivity was fairly low (14.8%). The reaction did not occur at all when only Lewis acid (ZnCl_2_) was used (table 1, entry 6), which indicates that Lewis acid itself has not the ability of hydrogenation catalysis. When Pd/AC and ZnCl_2_ were used together, a high conversion of 99.8% and a selectivity of 76.0% to TMCH were obtained (table 1, entry 2). The result clearly indicates that the co-catalyst, ZnCl_2_, can greatly inhibit the over-hydrogenation to increase the selectivity. When CuCl_2_ was used as a co-catalyst, the conversion of isophorone was very low (16.6%), which suggests that hydrogenation of isophorone to TMCH was also inhibited (table 1, entry 3). When AlCl_3_ was used as a co-catalyst, the conversion was 67.1%, and the selectivity was 15.7% (table 1, entry 4), both were lower than those of ZnCl_2_ as a co-catalyst. CrCl_3_ was further used as a co-catalyst, and it could increase the conversion to 74.3%, but the selectivity was low (only 19.0%) (table 1, entry 5). Among the four kinds of Lewis acids, CuCl_2_, AlCl_3_ and CrCl_3_ are relatively strong Lewis acids, which interact not only with C=O but also with C=C on isophorone, and inhibit the conversion. ZnCl_2_ is a relatively weak Lewis acid and shows the best performance.

**Figure 1.**
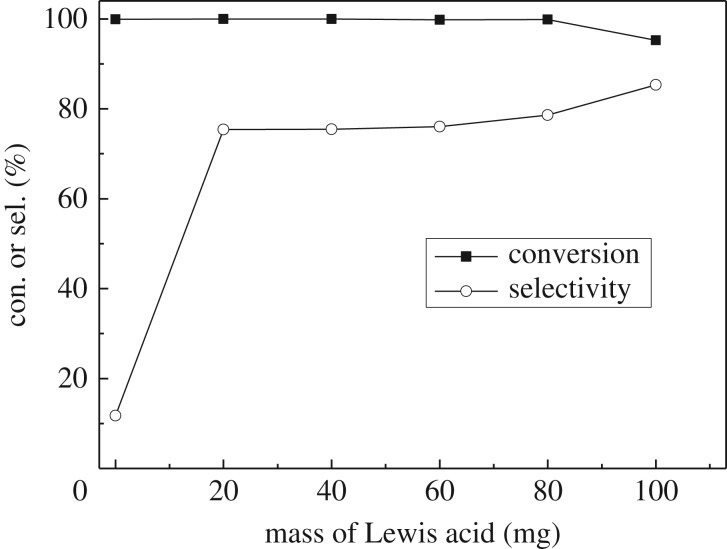
Effect of the amount of added ZnCl_2_ on the hydrogenation reaction of isophorone. Conditions: Pd/AC catalyst, 60 mg; Lewis acid, ZnCl_2_; initial pressure of H_2_, 2.00 MPa; reaction temperature, 100°C; time, 4 h.

**Table 1.** Hydrogenation of isophorone with different Lewis acids. Conditions: Pd/AC catalyst, 60 mg; Lewis acid, 60 mg; initial pressure of H_2_, 2.00 MPa; temperature, 100°C; time, 4 h. Dashes indicate that no Lewis acid was added.

entry	reactants	Lewis acids	conversion (%)	selectivity (%)
1	isophorone	—^a^	>99.9	14.8
2	isophorone	ZnCl_2_	99.8	76.0
3	isophorone	CuCl_2_	16.6	97.8
4	isophorone	AlCl_3_	67.1	15.7
5	isophorone	CrCl_3_	74.3	19.0
6	isophorone	ZnCl_2_^b^	0	0
7	TMCH	—^a^	87.2	—
8	TMCH	ZnCl_2_	25.2	—

^a^No Lewis acid was added.

^b^Only ZnCl_2_ was used (no Pd/AC).

Then the question arises whether the co-catalyst Lewis acid could protect C=O of TMCH and inhibit over-hydrogenation. The experiments of TMCH hydrogenation reaction were performed. The conversion of TMCH catalysed by Pd/AC and ZnCl_2_ is much lower than that catalysed only by Pd/AC (table 1, entries 7 and 8), which indicates the carbonyl on TMCH was protected by ZnCl_2_ against hydrogenation. It is noted that there is no other solvent used for the above hydrogenation reaction of isophorone to TMCH. Reasonably, the reactant isophorone and product TMCH serve as solvents, which promote the reaction rate.

Liu *et al*. [14] reported that Lewis acid not only interacts with C=O on cyclohexanone to inhibit the hydrogenation of cyclohexanone to cyclohexanol, but also coordinates with the benzene ring of phenol and makes it more active, which can promote the selective hydrogenation of phenol to cyclohexanone. Deshmukh *et al*. [15] also reported that Lewis acid could cooperatively interact with benzene resulting in a highly efficient catalytic hydrogenation of benzene under ambient conditions over Pd/AC. Our work indicates that Lewis acid also interacts with C=O on isophorone to inhibit the over-hydrogenation of isophorone to 3,3,5-trimethylcyclohexanol. Nevertheless, Lewis acid can also interact with C=C bond on isophorone and prohibit the hydrogenation of C=C bond on isophorone. Thus, the conversion rate was decreased. It is reported that Lewis acid can interact with benzene ring (one of the three C=C bonds) and make it unstable, resulting in activating aromatic compounds [15]. By contrast, isophorone has only one C=C bond, so Lewis acid possibly interacts with it and make it passivated to break.

The effect of amount of added ZnCl_2_ on the hydrogenation reaction was studied, and the results are shown in figure 1. It can be seen that when there is no ZnCl_2_, the selectivity to TMCH is very low (approx. 12%). When 20 mg of ZnCl_2_ (one-third amount of Pd/AC) was added to the reaction system, the selectivity increased greatly to 77%, while the conversion of isophorone kept the same value (100%). When the amount of ZnCl_2_ was further increased to 60 mg (equal to the amount of Pd/AC catalyst), the conversion and selectivity showed no obvious change. It means that a small amount of ZnCl_2_ is enough to inhibit the over-hydrogenation. However, when the amount of added ZnCl_2_ is increased to 100 mg, the selectivity to TMCH increases to a small extent, but the conversion of isophorone decreases, which indicates that too large an amount of ZnCl_2_ may passivate the C=C bond of isophorone, as discussed above.

**Figure 2.**
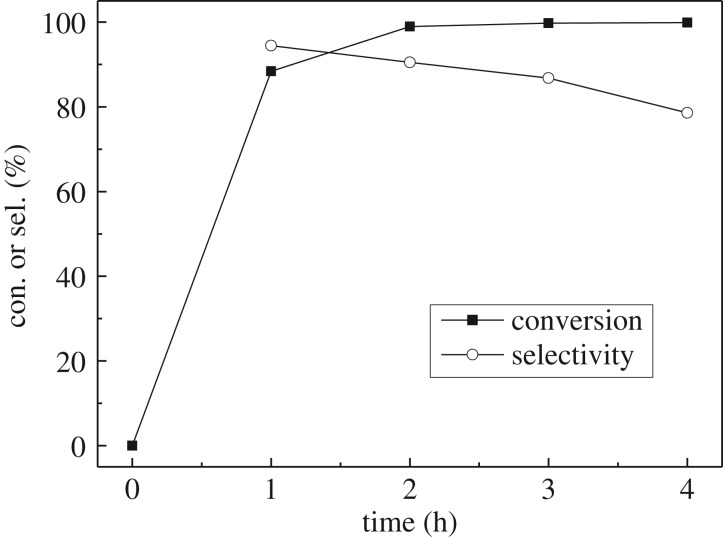
Effect of the reaction time on the hydrogenation reaction of isophorone. Conditions: Pd/AC catalyst, 60 mg; Lewis acid, ZnCl_2_, 60 mg; initial pressure of H_2_, 2.00 MPa; temperature, 100°C.

Figure 2 shows the influence of the reaction time on the conversion of isophorone and selectivity to TMCH. As expected, the conversion increases with time up to 100%. The selectivity at 1 h is 94%, and it decreases slowly with further increasing the reaction time. The hydrogenation of isophorone is a consecutive reaction, as shown in scheme 1. Too long reaction time will result in over-hydrogenation and production of 3,3,5-trimethylcyclohexanol.

It was reported that the presence of solvents had an influence on the hydrogenation of isophorone and it was difficult to achieve a high selectivity without using a solvent [16]. Therefore, we studied the effect of solvents, such as ethanol, dichloromethane and supercritical CO_2_, which are studied in the literature as effective solvents.

Table 2 shows the effect of solvents on the hydrogenation of isophorone with and without Lewis acid ZnCl_2_. When ethanol was used as the solvent without ZnCl_2_, the conversion of isophorone is more than 99.9% and the selectivity to TMCH is 73.8%. Compared with the results of only use of Pd/AC (table 1, entry 1), adding a solvent (not only ethanol but also dichloromethane and CO_2_) can obtain higher selectivity to TMCH (table 2, entries 1, 3 and 5), like additive ZnCl_2_.

**Table 2.** Hydrogenation of isophorone with or without ZnCl_2_ Lewis acid catalyst using different solvents. Conditions: Pd/AC catalyst, 60 mg; initial pressure of H_2_, 2.00 MPa; initial pressure of CO_2_ when supercritical CO_2_ was used as a solvent, 7.00 MPa (the total pressure could be increased to about 12 MPa during the reaction).

entry	mass of ZnCl_2_ (mg)	temp. (^o^C)	time (h)	solvents	conversion (%)	selectivity (%)
1	0	90	4	C_2_H_5_OH	>99.9	73.8
2	60	90	4	C_2_H_5_OH	>99.9	97.8
3	0	90	4	CH_2_Cl_2_	>99.9	97.6
4	60	90	4	CH_2_Cl_2_	98.9	98.0
5	0	90	4	CO_2_	97.6	99.0
6	60	90	4	CO_2_	91.2	99.8
7	60	100	4	CO_2_	93.7	99.3
8	60	90	6	CO_2_	97.1	99.3
9	120	30	22	CH_2_Cl_2_	99.6	99.3
10	120	40	22	CH_2_Cl_2_	99.7	98.3
11	120	50	22	CH_2_Cl_2_	99.9	97.1
12	120	60	22	CH_2_Cl_2_	99.9	95.4
13	60	60	22	CH_2_Cl_2_	75.4	97.3

Interestingly, when Lewis acid ZnCl_2_ and solvent ethanol were used together, the selectivity was 97.8% (table 2, entry 2), which is better than using ethanol or ZnCl_2_ separately. When ZnCl_2_ and dichloromethane were used together, the conversion decreased to 98.9% from 99.9% using only dichloromethane as solvent, while the selectivity slightly increased to 98.0% from 97.6%. The results indicate that both dichloromethane and ZnCl_2_ can synergistically increase the selectivity to TMCH, but they may inhibit the hydrogenation of C=C on the molecular structure of isophorone, which results in a slow reaction rate and a low conversion of isophorone.

Supercritical CO_2_ can also be seen as a weak Lewis acid and it has the characteristic advantages of waste minimization, easy product separation and pressure tunability [17–19]. Therefore, supercritical CO_2_ is broadly considered as a solvent in chemical reaction and separation [20]. Hitzler *et al*. reported the highly selective hydrogenation of isophorone to TMCH with an aminopolysiloxan-supported palladium in supercritical CO_2_ [21]. Sato *et al*. also used supercritical CO_2_ as a solvent for improving the selectivity of hydrogenation of isophorone [7]. In this work, we also used supercritical CO_2_ as a solvent for the hydrogenation of isophorone. Supercritical CO_2_ has a similar effect as dichloromethane. When supercritical CO_2_ and ZnCl_2_ were used together, the selectivity was increased to 99.8%, but the conversion decreased to 91.2% (table 2, entries 5 and 6). When the temperature or the reaction time was further increased, the conversions were increased to 93.7% and 97.1%, respectively (table 2, entries 7 and 8). Rode *et al*. reported that a supported Pd catalyst is less active for the ring hydrogenation of phenol under the supercritical CO_2_ condition than that in the liquid-phase condition [22]. This may be similar to the isophorone hydrogenation reaction in which the conversion rate of isophorone under supercritical CO_2_ conditions is lower than that of ethanol or dichloromethane.

Increasing the reaction temperature can accelerate the rate of the reaction, but it can also reduce the selectivity (table 2, entries 9–12) because the inhibition of Lewis acid will be weakened at high temperature [14]. At the same time, the acid–base interaction between Lewis acid and cyclohexanone inhibits further hydrogenation to cyclohexanol. Hence, by tuning temperature and reaction time, the yield of TMCH can be greater than 99%.

When organic solvents and supercritical CO_2_ were used in the reaction system, they could dissolve the reactants and product to form a solution, which could disperse the catalyst and Lewis acid under stirring. Moreover, organic solvents and high-pressure CO_2_ could increase the diffusion coefficient of the reactants and products. Products were easily removed from the reactive sites, avoiding the over-hydrogenation of TMCH and increasing the selectivity.

**Figure 3.**
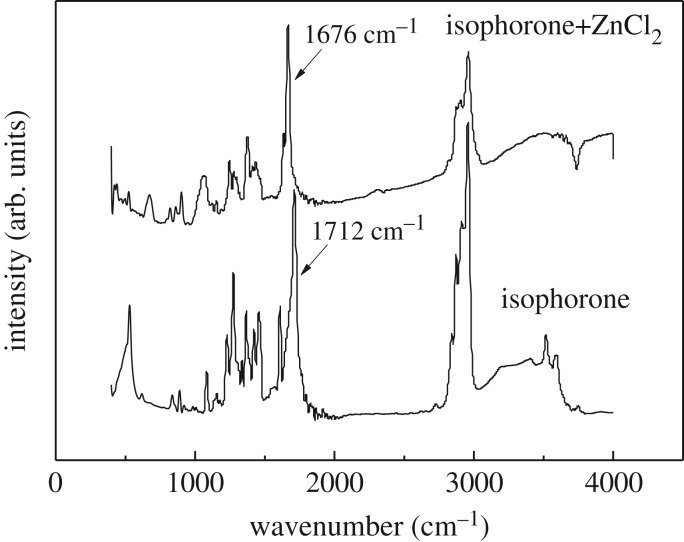
The infrared spectra of isophorone with and without ZnCl_2_.

**Figure 4.**
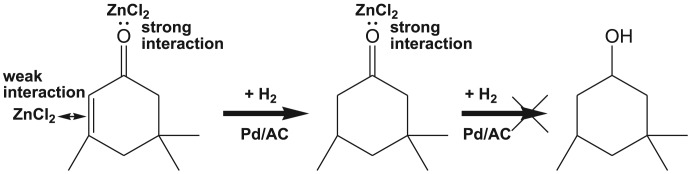
A possible mechanism of the selective hydrogenation of isophorone to TMCH by Lewis acid ZnCl_2_.

Liu *et al*. [14] suggested that Lewis acid had a complexation on the oxygen lone pair of electrons of the carbonyl group, thereby hindering attack from the active hydrogen. We also observed the interaction between TMCH and a Lewis acid such as ZnCl_2_, as shown in figure 3. The absorption bond of the C=O stretching vibration shifts from 1712 cm^−1^ in the absence of ZnCl_2_ to 1676 cm^−1^ in the presence of ZnCl_2_, but no other obvious differences were found. It was reported that this shift was consistent with coordination of the Lewis basic C=O group to the Lewis acid [14,23]. Similarly, Lewis acid may have some role with the electron cloud surrounding the C=C bond, but this complexation strength seems to be far weaker than that between the Lewis acid and the carbonyl group. It was found that ZnCl_2_ could strongly inhibit the hydrogenation of TMCH, demonstrating a strong interaction between ZnCl_2_ and C=O on isophorone. Therefore, a proposed mechanism of hydrogenation of isophorone with Lewis acid ZnCl_2_ is shown in figure 4.

## 4. Conclusion

In summary, the inhibition of TMCH hydrogenation is a key factor for improving the selectivity of hydrogenation of isophorone to TMCH. Lewis acid was found to protect TMCH and efficiently inhibit TMCH hydrogenation, but has some inhibitory effect on isophorone hydrogenation to TMCH. Therefore, among several Lewis acids, ZnCl_2_ was chosen to improve the selectivity of the reaction and to keep the high hydrogenation rate of isophorone. It was found that solvents like ethanol, dichloromethane and supercritical CO_2_ could play a role similar to Lewis acid in the reaction. Together, Lewis acid and solvent could synergistically improve both the conversion rate and selectivity to more than 99%. The mechanism of Lewis acid inhibition was proposed.

## Data accessibility

This article does not contain any additional data.
